# Supplementary material for: Perceptions of Tailored Dietary Advice to Improve the Nutrient Adequacy of the Diet in French Pregnant Women
Source: Nutrients. 2021 Dec 25;14(1):85. doi: 10.3390/nu14010085 (PMC8747067; doi:10.3390/nu14010085)
Supplement: Supplementary file 1 [file nutrients-14-00085-s001.zip › nutrients-1519817-supplementary.pdf]

Supplementary Materials

File S1. Details of the study relative to the research team and reflexivity, study design and analysis and findings according to the consolidated criteria for reporting qualitative research (COREQ) [35].

Domain 1: Research team and reflexivity

Personal characteristics

The first author (CB), a PhD student whose research interest is nutrition during pregnancy, was the moderator. She was chosen as she was a young woman, but not a healthcare professional, to facilitate discussions by the participants and to limit any judgmental feelings. She had previously been trained in leading focus group interviews.

Relationship with participants

The moderator did not establish a relationship with the participants prior to the study. However, she had a brief telephone interview with each participant at the time of recruitment in order to describe the content of focus group sessions and briefly present herself (credentials, occupation, research interests and reasons for performing the research).

Domain 2: Study design

Theoretical framework

As no previous study had been performed on the eating behaviors of French pregnant women, we did not state a pre-determined theory before data collection. An inductive thematic approach, adapted from the grounded theory, was therefore implemented to analyse the data. This approach involves familiarization with the data, an open-coding process, and data interpretation in the themes derived from identified codes [36, 37].

Participant selection

In Paris, pregnant women were recruited from consumers registered in the database of a generalist market research company. The company contacted women living in the Paris region by email, and if they were pregnant they were invited to take part in a focus group session on diet and nutrition. Those interested in participating registered with the company using an online form. In Aix-en-Provence, pregnant women were recruited using posters displayed in the gynaecologists' waiting room in a maternity clinic (L'Etoile Maternity Clinic, Aix-en-Provence, Provence Alpes Côte d'Azur, France). They could register either by email or by calling the first author. After registering, the women were contacted by telephone by the first author in order to verify their eligibility regarding the inclusion criteria and to arrange their attendance at one of the scheduled sessions. Before each session, the first author described the context, objectives and content of the focus group session, giving information on the time, date, duration and location of the session and answering any questions about the study. In order to anticipate the drop-outs that can affect attendance at focus groups, even after registration, at least eight participants were scheduled for each session. Finally, a total of 61 pregnant women were scheduled, 21 dropped out that resulted in a total of 40 pregnant women participated in the study.

Setting

Each focus group session was organized in a special meeting room at the maternity clinic in Aix-en-Provence or at AgroParisTech School of Life Sciences in Paris. Only the moderator, assistant moderator and participants were present during a session. Healthy snacks and drinks were offered to the participants.

Data collection

Details relative to the interview guide, number of interviews, visual recordings, field notes and durations are given in the 'Methods' section of the article.

Following each focus group session, the moderator and assistant moderator debriefed the session together, identified the main themes to be used for the coding process and wrote a report. Three focus groups were initially carried out and transcribed. A preliminary analysis was made by the first and last authors regarding the reports on the first three first sessions, so as to identify whether thematic saturation had been reached or if the interview guide needed to be adapted because of emerging themes. They agreed to perform four more focus group sessions without modifying the interview guide. A further analysis was performed by the first and last authors, which covered the reports on all seven sessions. The first and last authors agreed that the saturation had been reached, inasmuch as no new themes had been identified by this point. A short summary of the findings was sent by email to all the participants.

Domain 3: Analysis and findings

Data analysis

Details relative to the number of data coders and the software used are described in the 'Methods' section of the article. Details relative to the description and derivation of themes and sub-themes are provided in the Additional file 3. After completion of the analysis, a short summary of the findings was sent to the participants by email.

Reporting

The themes and sub-themes are presented in the 'Results' section of the article. Quotations from the participants were used to illustrate the themes.

Table S1. Themes and subthemes identified in the focus group study.

Themes	Sub-themes
**The ideal dietary advice tool for pregnant women**	**1. General features:**<br>- A tailored, easy-to-use and not guilt-inducing tool<br>- A nine-month support<br>**2. Content**<br>**3. User profile**
**The tailored dietary advice tool we suggested with our model**	**1. General feelings about this tool**<br>- Strengths<br>- Weaknesses<br>**2. Specific feelings about each type of dietary changes**<br>- Tailored dietary changes of type 1<br>- Tailored dietary changes of type 2<br>- Tailored dietary changes of type 3<br>**3. Ideas for improvement**

Table S2. Answers to the final questionnaire (n=80) designed through an adaptation of the Theory of Planned Behavior.

**Intention**	Since I am pregnant, I have intended to modify my dietary habits	
	All the time	23.8% (19)[1]
	From time to time	71.3% (57)
	Rarely	2.5% (2)
	Never	2.5% (2)
**Attitudes**	To modify my dietary habits is good for my health	

	Strongly agree	53.8% (43)
	Agree	43.8% (35)
	Neither/Nor agree	2.5% (2)
	Disagree	0.0% (0)
	Strongly disagree	0.0% (0)
	To modify my dietary habits is good for my baby's health	
	Strongly agree	72.5% (58)
	Agree	23.8% (19)
	Neither/Nor agree	3.8% (3)
	Disagree	0.0% (0)
	Strongly disagree	0.0% (0)
	To modify my dietary habits is good for increasing my vitamins and minerals intakes	
	Strongly agree	42.5% (34)
	Agree	48.8% (39)
	Neither/Nor agree	8.8% (7)
	Disagree	0.0% (0)
	Strongly disagree	0.0% (0)
**Subjective norm**	People close to me advise me to modify my dietary habits	
	Strongly agree	20.0% (16)
	Agree	23.8% (19)
	Neither/Nor agree	38.8% (31)
	Disagree	11.3% (9)
	Strongly disagree	5.0% (4)
	No answer	1.3% (1)
	My healthcare providers during pregnancy advise me to modify my dietary habits	
	Strongly agree	33.8% (27)
	Agree	37.5% (30)
	Neither/Nor agree	13.8% (11)
	Disagree	8.8% (7)
	Strongly disagree	6.3% (5)
**Perceived Behavioral Control**	To modify my dietary habits is difficult because it would take too much time to me	
	Strongly agree	7.5% (6)
	Agree	16.3% (13)
	Neither/Nor agree	18.8% (15)
	Disagree	36.3% (29)
	Strongly disagree	21.3% (17)
	To modify my dietary habits is difficult because it would cost too much to me	
	Strongly agree	10.0% (8)
	Agree	28.8% (23)
	Neither/Nor agree	20.0% (16)
	Disagree	23.8% (19)
	Strongly disagree	16.3% (13)
	No answer	1.3% (1)
	To modify my dietary habits is difficult because all foods I want to buy are not necessarily available	
	Strongly agree	10.0% (8)
	Agree	28.8% (23)
	Neither/Nor agree	20.0% (16)
	Disagree	23.8% (19)
	Strongly disagree	17.5% (14)
	To modify my dietary habits is difficult because I don't like eating some foods	
	Strongly agree	23.8% (19)

	Agree	43.8% (35)
	Neither/Nor agree	11.3% (9)
	Disagree	10.0% (8)
	Strongly disagree	11.3% (9)
	To modify my dietary habits is difficult because of digestive disorders related to pregnancy	
	Strongly agree	12.3% (10)
	Agree	19.8% (16)
	Neither/Nor agree	12.3% (10)
	Disagree	18.5% (15)
	Strongly disagree	35.8% (29)

[1] Percentage of respondants (number of respondants), all such values.
